# Very Late Chronic Total Occlusion After Drug-Coated Balloon Treatment for a Diffuse Long Coronary Lesion in Unstable Angina: A Case Report

**DOI:** 10.7759/cureus.115481

**Published:** 2026-08-31

**Authors:** Yu Sugawara

**Affiliations:** 1 Cardiovascular Medicine, Yamato Kashihara Hospital, Kashihara, JPN

**Keywords:** cardiac computed tomography (ct), case report, chronic total occlusion (cto), drug-coated balloon, intravascular ultrasound (ivus), percutaneous coronary intervention

## Abstract

A drug-coated balloon (DCB) strategy has become an established option for de novo coronary lesions, offering the advantage of avoiding permanent implants. However, very long-term vascular changes after stentless percutaneous coronary intervention (PCI) remain insufficiently characterized. We report a case of chronic total occlusion that developed eight years after DCB angioplasty for a diffuse long right coronary artery lesion in a patient with unstable angina.

A 65-year-old man developed exertional chest discomfort approximately seven years after the index procedure, and cardiac computed tomography demonstrated severe calcification and suspected occlusion of the previously treated segment. At the index procedure, we selected a DCB-only strategy because intravascular ultrasound (IVUS) revealed a long, diffuse lesion requiring multiple stents and extensive drug-eluting stent (DES) implantation carries increased long-term risks. During repeat PCI, IVUS demonstrated dense calcification and high-echoic crescentic neointimal tissue without imaging features suggestive of thrombus. The imaging findings suggested atherosclerotic progression, potentially compounded by the limited acute luminal gain achieved at the index procedure, the cumulative burden of cardiovascular risk factors, and chronic inflammatory cell infiltration. Although the pharmacological effect of paclitaxel diminishes within months, preclinical studies have suggested that low residual tissue concentrations might contribute to late fibrotic or calcific remodeling. Device-related neoatherosclerosis appears unlikely because of the absence of a metallic scaffold.

This case illustrates the multifactorial nature of very late vascular changes after stentless PCI and demonstrates how multimodality imaging, including cardiac computed tomography and IVUS, can describe late lesion morphology and healing patterns. The findings highlight the complex late-phase vascular morphology that may emerge in patients with diffuse disease and multiple risk factors and underscore the clinical value of comprehensive imaging assessment at very late follow-up.

## Introduction

Drug-coated balloons (DCBs) are widely used to treat de novo coronary artery lesions, which are native lesions without prior treatment. These devices deliver antiproliferative drugs to the vessel wall without leaving a permanent implant, an approach that avoids long-term biological responses associated with permanent metallic stents. Paclitaxel-coated balloons have long been the standard, and sirolimus-coated balloons have recently become available. Current evidence indicates that sirolimus-coated balloons are non-inferior to paclitaxel-coated balloons [1]. In appropriate clinical settings such as stable de novo lesions, particularly small vessels and patients at high bleeding risk, both types of DCBs are comparable to drug-eluting stents (DES) [2]. DCBs offer the advantage of avoiding stent-related complications because no permanent scaffold remains in the coronary artery. Reported benefits include late lumen enlargement [3], a phenomenon characterized by progressive luminal expansion, and the absence of a sustained drug reservoir in the chronic phase [4]. In contrast, DES may experience late failure due to chronic inflammation, atherosclerotic change within the stented segment, progression of native coronary atherosclerosis, or adverse vascular remodeling associated with the permanent implant [5]. These considerations provide the clinical context for the present case. We describe a rare instance of chronic total occlusion (CTO) identified eight years after DCB treatment, evaluated with cardiac computed tomography (CT), a non-invasive modality that visualizes coronary anatomy, and intravascular ultrasound (IVUS), which provides cross-sectional imaging of the vessel wall.

## Case presentation

A 65-year-old man with diabetes mellitus, hypertension, and dyslipidemia, receiving vildagliptin 100 mg, metformin 1000 mg, amlodipine 5 mg, rosuvastatin 2.5 mg, and aspirin 100 mg with no family history of coronary artery disease and a non-smoking status, presented with exertional angina and was admitted to our hospital. On admission, his blood pressure was 130/80 mmHg, heart rate 60 beats per minute, and oxygen saturation 97% on room air, and he was afebrile. He appeared comfortable at rest. Cardiac rhythm was regular, no murmurs were audible, breath sounds were clear bilaterally, and there was no peripheral edema. He had previously been treated for unstable angina at 57 years of age. At that time, his blood pressure was 142/80 mmHg, heart rate 71 beats per minute, and oxygen saturation 100% on room air, and he was afebrile. He appeared in no distress. Cardiac rhythm was regular, no murmurs were heard, breath sounds were clear bilaterally, and there was no peripheral edema or cyanosis. The electrocardiogram (ECG) demonstrated sinus rhythm, with no ST-segment changes (Figure 1).

**Figure 1:**
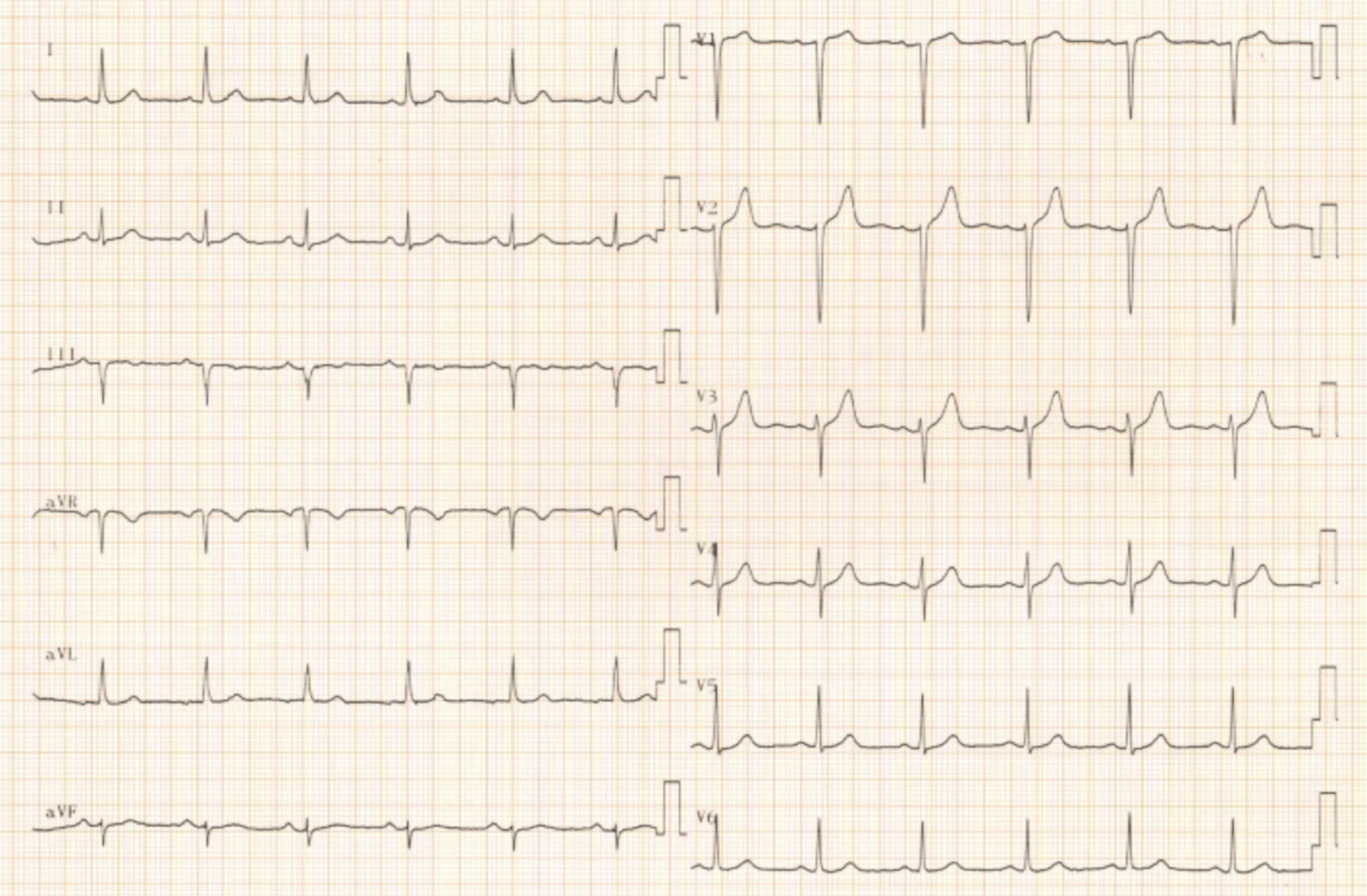
The ECG obtained at the index PCI showed sinus rhythm and no ST-segment changes ECG: electrocardiogram; PCI: percutaneous coronary intervention

His symptoms improved after percutaneous coronary intervention (PCI), and he was discharged three days later after a short period of inpatient observation considering the acute coronary syndrome. Aspirin 100 mg, clopidogrel 75 mg (clopidogrel was discontinued one year after in accordance with current guideline recommendations [6]), rosuvastatin 2.5 mg, amlodipine 5 mg, vildagliptin 100 mg, and metformin 1000 mg were prescribed during the follow-up, with reported good adherence. The outcome was favorable without chest discomfort. Therefore, he did not wish to undergo a coronary assessment. During the follow-up period, the patient's blood pressure was 120-130/70-80 mmHg, hemoglobin A1C level 6.0-7.0%, and low-density lipoprotein (LDL) cholesterol level 50-70 mg/dL. Although his serum creatinine was 0.82 mg/dL and the estimated glomerular filtration rate (eGFR) was 76 mL/min/1.73 m² at the index procedure, at the second PCI, his serum creatinine had increased to 1.40 mg/dL, and the eGFR had decreased to 41 mL/min/1.73 m². Cardiac troponin T levels were normal, and the left ventricular ejection fraction was 65% without asynergy. Coronary angiography (CAG) revealed 99% stenosis of the middle segment of the right coronary artery (RCA) (Figure 2a, white arrowhead, Video 1). The reference vessel diameter was 2.5 mm, and a 2.5 mm balloon was selected to achieve a 1:1 balloon-to-artery ratio, in accordance with IVUS-guided balloon sizing. After predilatation with a 2.5 × 15 mm plain balloon, no flow limitation, thrombus, major dissection, or residual stenosis was observed. The lesion was then treated with SeQuent Please (B. Braun, Melsungen, Germany) 2.5 × 30 mm DCB inflated for 60 seconds (Figure 2b, white arrowheads). The final angiogram demonstrated Thrombolysis In Myocardial Infarction (TIMI) grade 3 flow (Figure 2c, Video 2) without flow-limiting dissection or thrombus.

**Figure 2:**
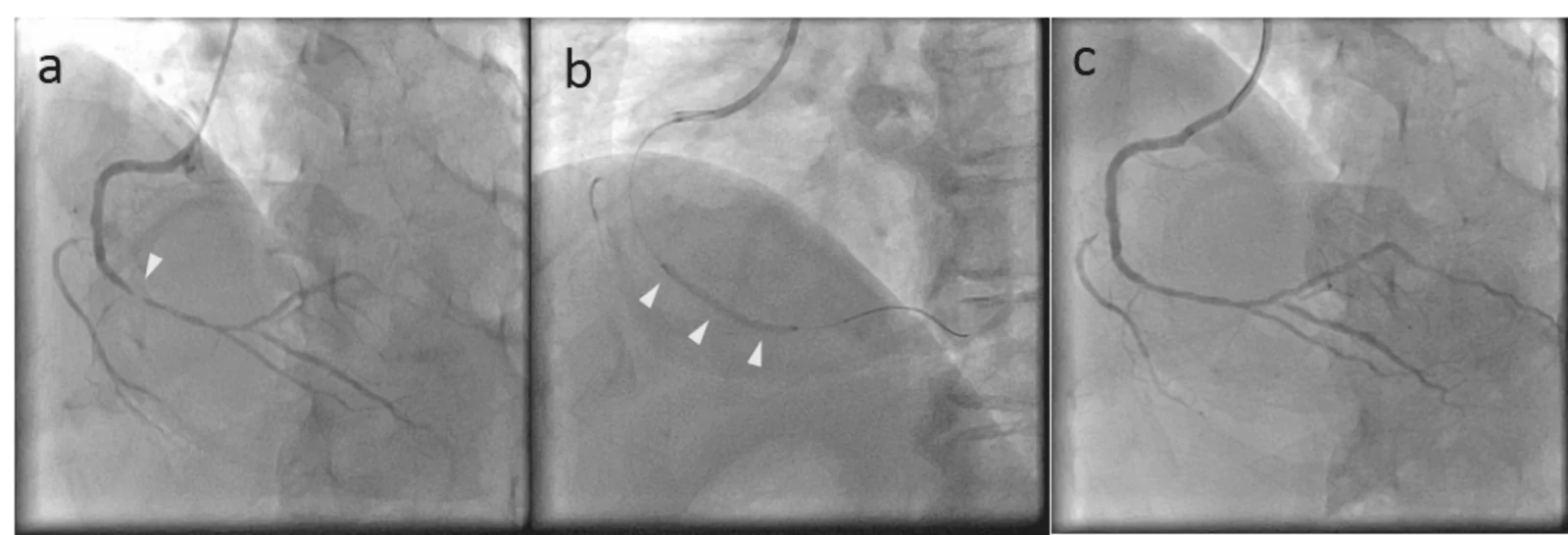
Right CAG at the index PCI (a) Right CAG showed severe stenosis in the mid-RCA (white arrowhead). (b) The lesion was dilated with a 2.5 × 30 mm paclitaxel‑coated balloon (white arrowheads). (c) Final CAG demonstrated TIMI grade 3 flow without flow limitation. CAG: coronary angiography; TIMI: Thrombolysis In Myocardial Infarction; PCI: percutaneous coronary intervention; RCA: right coronary artery

**Video 1:** Right CAG at the index PCI CAG demonstrated severe stenosis in the mid-RCA. CAG: coronary angiography; PCI: percutaneous coronary intervention; RCA: right coronary artery

**Video 2:** The final angiography at the index PCI showed adequate coronary flow with no residual stenosis PCI: percutaneous coronary intervention

IVUS demonstrated mixed-echogenicity plaque with echolucent and hyperechoic components (Figure 3a-3b). The luminal diameter at the culprit site measured 1.92 × 1.75 mm, the minimal luminal area (area bounded by the luminal border) was 2.67 mm², and the plaque burden (plaque‑plus‑media area/external elastic membrane (EEM) area) was approximately 66.7%.

**Figure 3:**
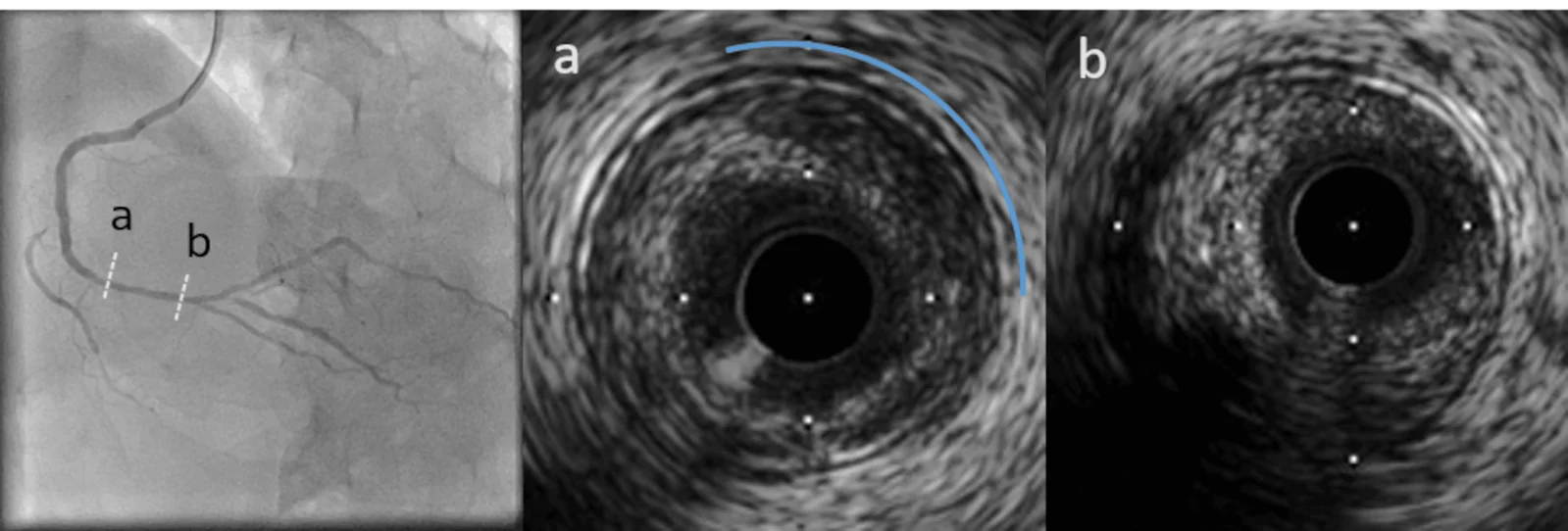
Cross-sectional IVUS images after drug-coated balloon angioplasty at the proximal (a) and distal (b) sites (a) Cross‑sectional IVUS image at the proximal site showed echolucent plaque (blue arc). The lumen diameter was 1.92 × 1.75 mm, lumen area was 2.67 mm^2^, and plaque burden was 66.7%. (b) Cross‑sectional IVUS image at the distal site showed echolucent plaques. The lumen diameter was 1.77 × 1.68 mm and lumen area was 2.23 mm^2^. IVUS: intravascular ultrasound

Nevertheless, seven years and five months after the DCB angioplasty, the patient experienced chest pain on exertion. Cardiac CT revealed heavy calcification in the mid-RCA (Figure 4a, white arrowheads). Additionally, severe stenosis or occlusion was suspected in the treated lesions (Figure 4b, white arrowheads).

**Figure 4:**
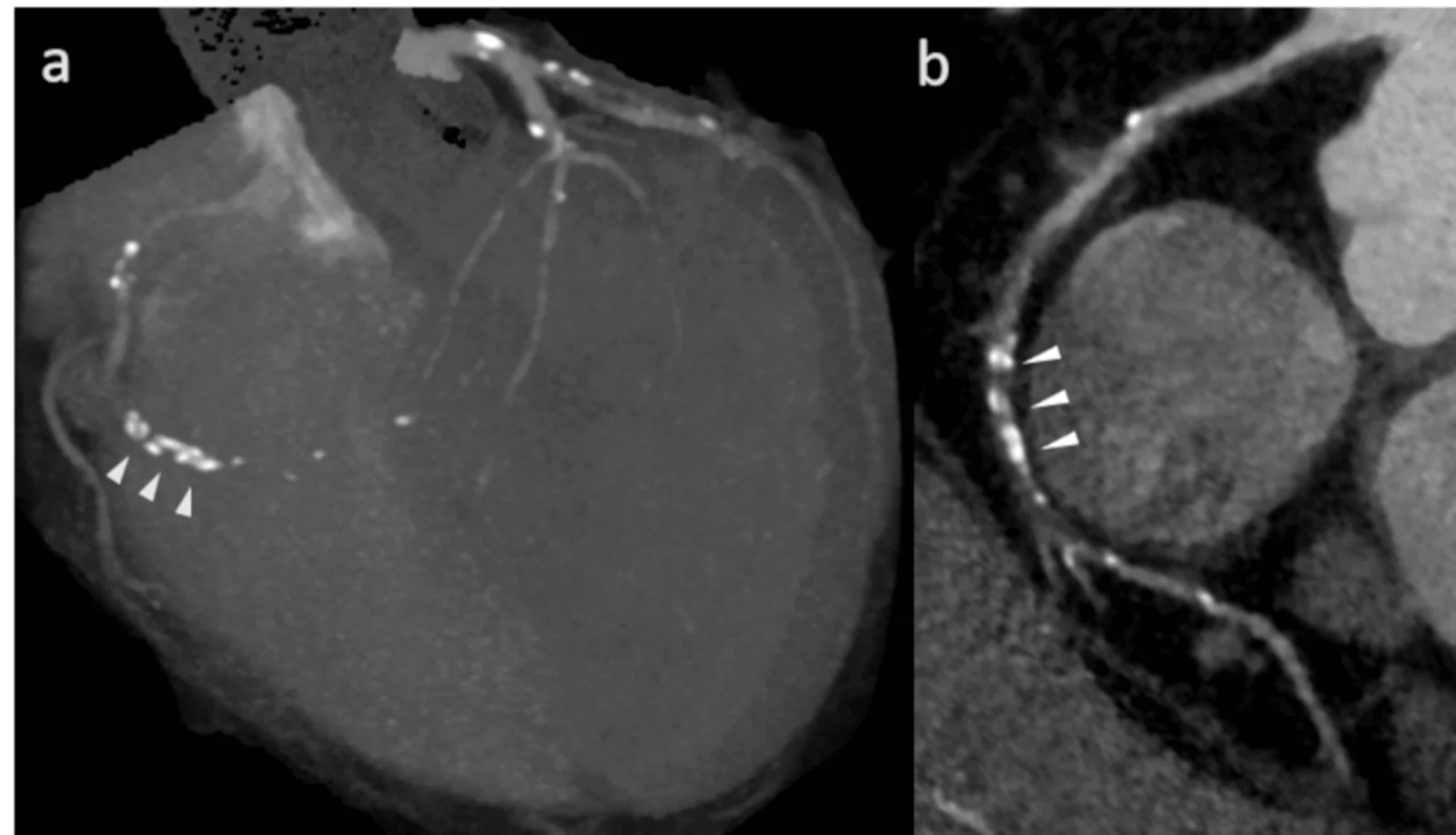
Cardiac CT findings seven years and five months after percutaneous coronary intervention (a) Cardiac CT revealed calcification in the mid-RCA (white arrowheads). (b) Cardiac CT demonstrated stenosis or occlusion with calcified plaque in the mid-RCA (white arrowheads). CT: computed tomography; RCA: right coronary artery

ECG at the second chest event revealed sinus rhythm and no ST-segment changes in any leads (Figure 5).

**Figure 5:**
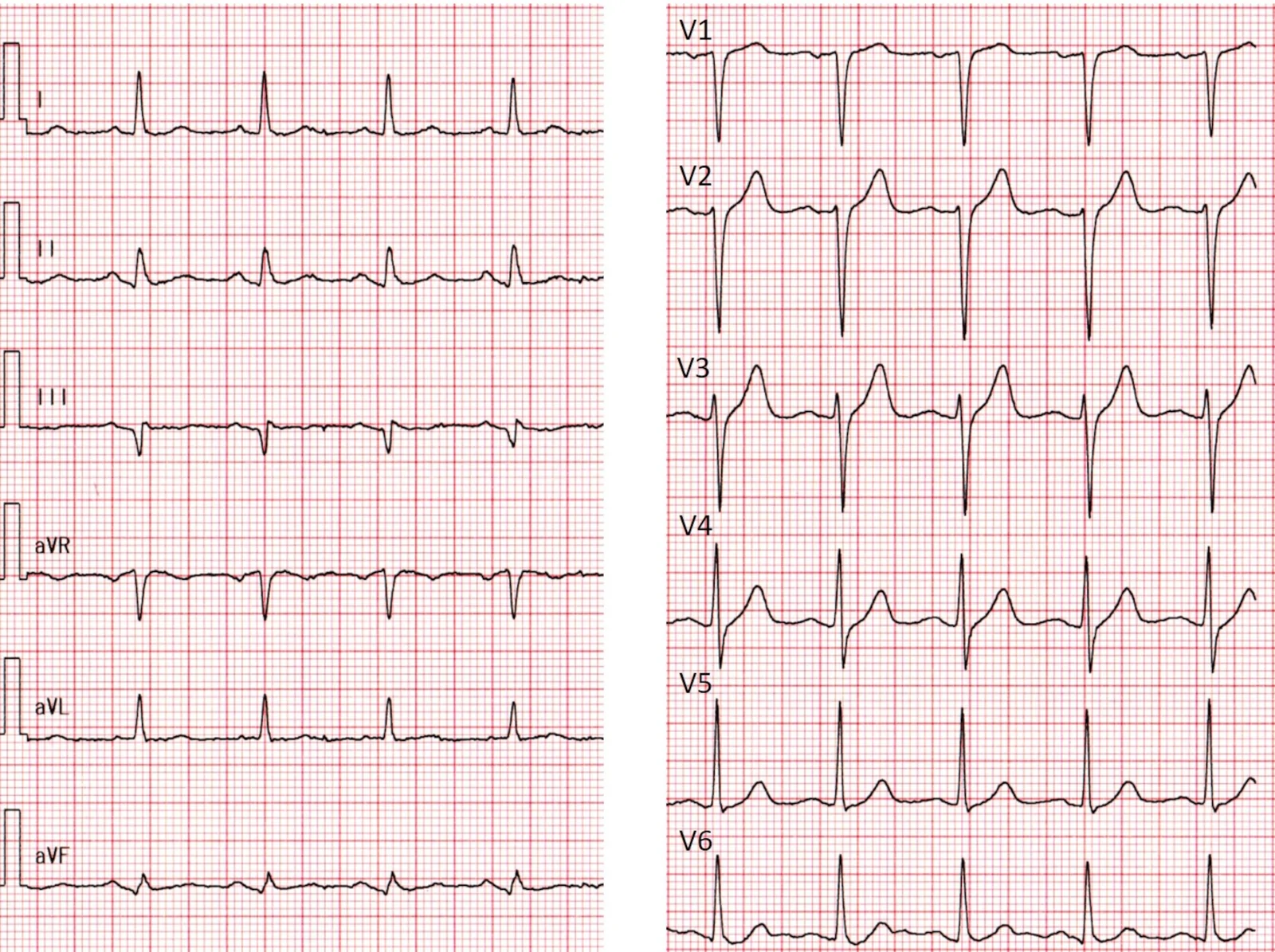
ECG at the time of the second chest event ECG revealed sinus rhythm and no ST-segment changes in any leads. ECG: electrocardiogram

Transthoracic echocardiography demonstrated a left ventricular ejection fraction of 55% with inferior wall hypokinesis and no myocardial thinning. At the time of the CTO presentation, laboratory tests showed a hemoglobin A1c of 6.9%, an LDL cholesterol of 65 mg/dL, a high‑density lipoprotein cholesterol of 60.8 mg/dL, a creatinine of 1.4 mg/dL, and an eGFR of 41 mL/min/1.73 m². Based on his symptoms and CT findings, CAG was performed eight years after the index procedure, as his symptoms were intermittent and mild rather than requiring immediate angiography after cardiac CT.

Angiography revealed total occlusion of the mid-RCA (Figure 6a, white arrowhead, Video 3), with a Japan-Chronic Total Occlusion score (J-CTO score) of 2, based on severe calcification and a lesion length of >20 mm. The left anterior descending and circumflex arteries showed mild atherosclerotic progression compared with the index angiogram. Using a 6F AL 0.75 guiding catheter, the GAIA‑1 (Asahi Intecc, Akatsuki‑cho, Japan) guidewire was advanced into the intra‑plaque space, followed by dilatation with a 2.5 × 10 mm plain balloon, which restored coronary flow (Figure 6b).

**Figure 6:**
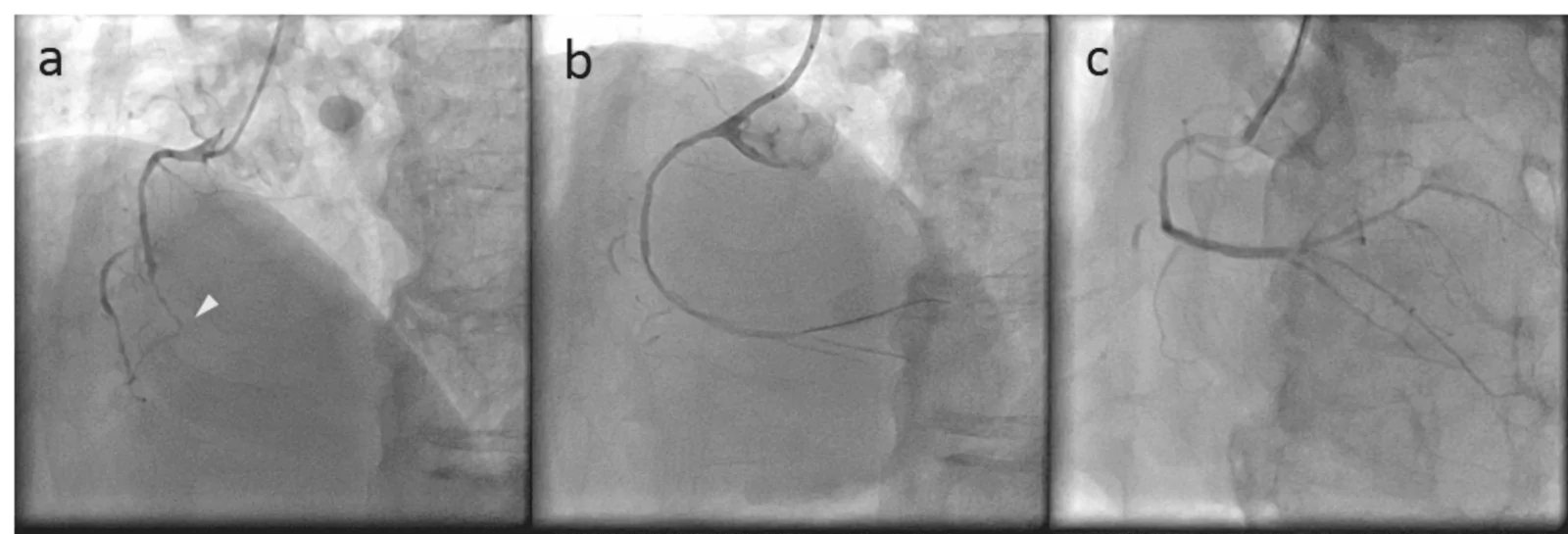
CAG at eight years after the first PCI (a) CAG revealed total occlusion of the mid-RCA (white arrowhead). (b) Recanalization of the RCA was achieved after dilatation with a 2.5 mm plain balloon. (c) Final CAG after the implantation of a 2.5 × 38 mm drug-eluting stent demonstrated good coronary flow without residual stenosis. CAG: coronary angiography; PCI: percutaneous coronary intervention; RCA: right coronary artery

**Video 3:** CAG at eight years after the first PCI CAG performed eight years after the initial PCI revealed total occlusion of the mid-RCA. CAG: coronary angiography; PCI: percutaneous coronary intervention; RCA: right coronary artery

After flow restoration, IVUS was performed, and the panels "a" and "b" in Figure 7 correspond to those in Figure 3. IVUS revealed calcification (Figure 7a, orange arrows) with dissection (Figure 7a, blue arrow) at the former culprit site and a crescentic intimal tissue with calcification (Figure 7b, orange dots, blue arc, Video 4) overlying the residual plaque at the distal site.

**Figure 7:**
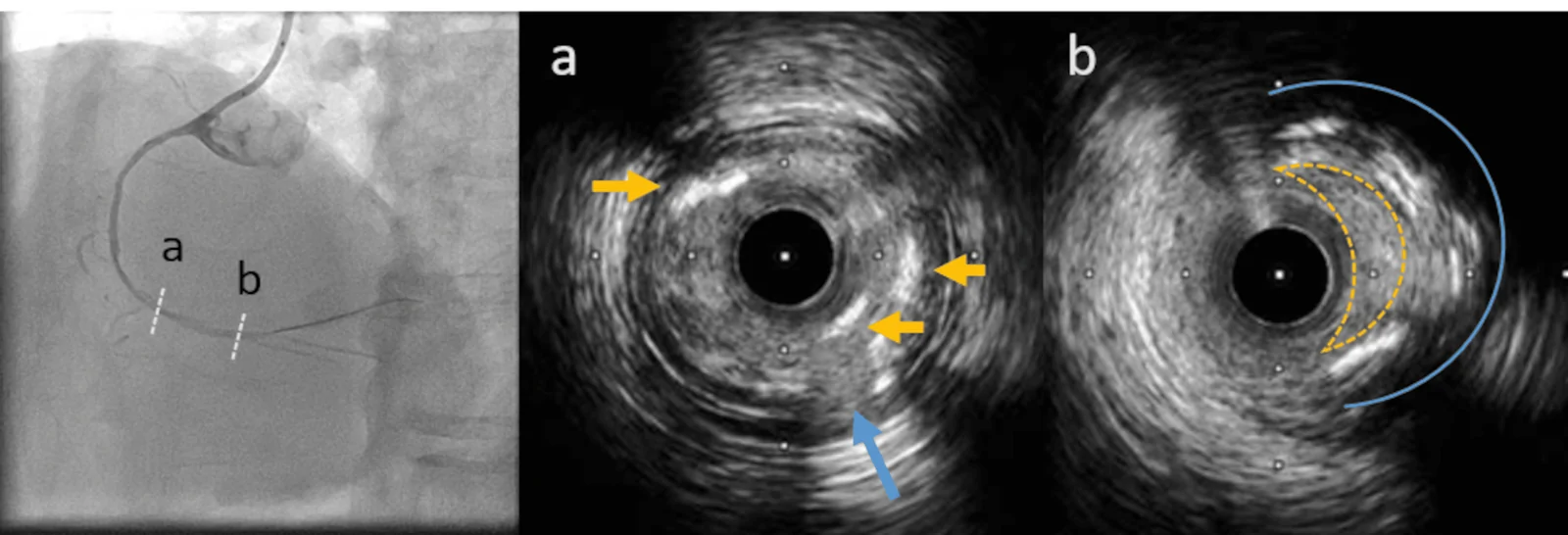
IVUS images of the lesion at the proximal (a) and distal (b) sites (a) Cross‑sectional IVUS at the proximal site showed high‑echoic intimal thickening with calcifications (orange arrows) and a dissection (blue arrow). (b) Cross‑sectional IVUS at the distal site revealed high‑echoic crescent‑shaped intimal thickening (orange crescent‑shaped dots) on the residual plaque and calcifications (blue arc). IVUS: intravascular ultrasound

**Video 4:** IVUS findings at the CTO‑PCI after 2.5 mm plain balloon dilatation IVUS revealed calcification and high-echoic neointimal proliferation. IVUS: intravascular ultrasound; CTO: chronic total occlusion; PCI: percutaneous coronary intervention

A XIENCE Skypoint (Abbott, Santa Clara, CA, USA) 2.5 × 38 mm DES was implanted. The final CAG demonstrated good coronary flow (Figure 6c, Video 5).

**Video 5:** Final CAG after stent deployment The final CAG demonstrated good coronary flow following the implantation of an everolimus-eluting stent. CAG: coronary angiography

After stent implantation, IVUS was performed, and the panels "a" and "b" in Figure 8 correspond to those in Figure 7. Cross-sectional IVUS (Figure 8a-8b) indicated that the diameter passing through the center of the stent (stent diameter) was 2.50 × 2.29 mm and the cross‑sectional area bounded by the inner surface of the stent struts (stent area) was 4.28 mm^2^ at the previously treated site (Figure 8a).

**Figure 8:**
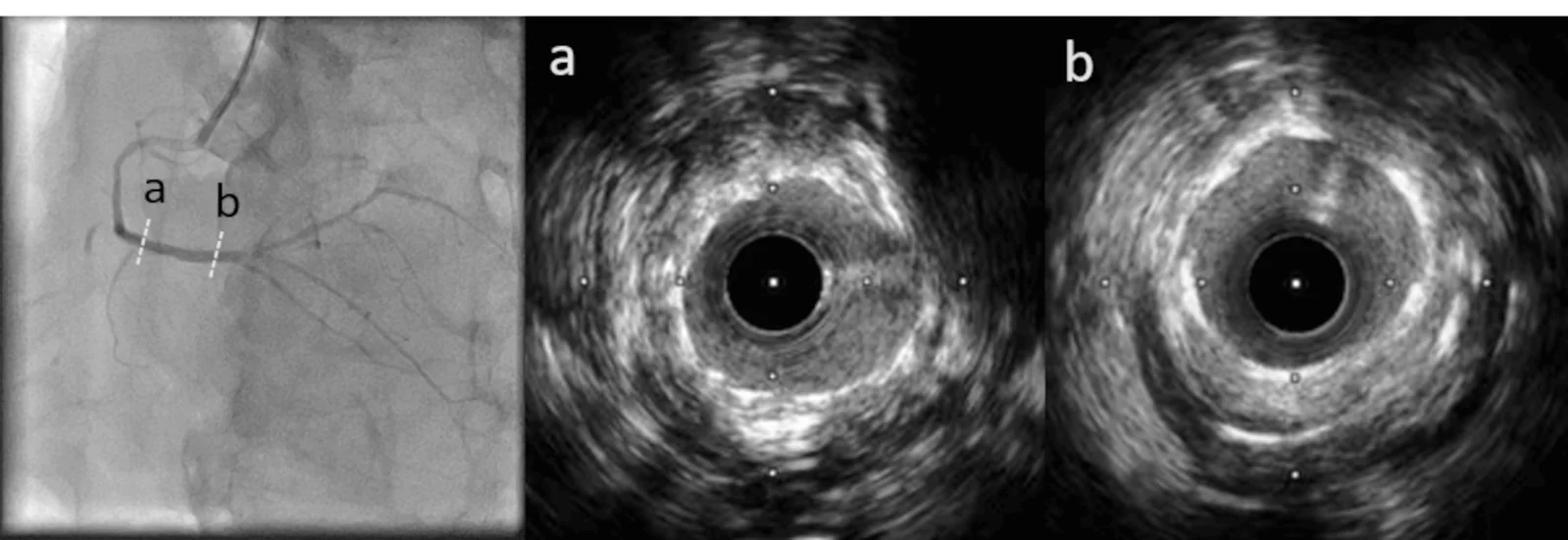
IVUS images of the lesion treated with a drug-eluting stent at the proximal (a) and distal (b) sites (a) Cross‑sectional IVUS at the proximal site demonstrated adequate stent expansion. The stent diameter measured 2.50 × 2.29 mm, and the stent area was 4.28 mm². (b) Cross‑sectional IVUS at the distal site also showed adequate stent expansion. The stent diameter measured 2.45 × 2.35 mm, and the stent area was 4.31 mm². IVUS: intravascular ultrasound

The patient was discharged one day after PCI. Following DES implantation, regular clinical follow‑up was arranged, and dual antiplatelet therapy consisting of aspirin 100 mg and clopidogrel 75 mg was resumed. Rosuvastatin 2.5 mg, ezetimibe 10 mg, amlodipine 5 mg, vildagliptin 100 mg, and metformin 1000 mg were also administered, and his self-reported medication adherence was good. After discharge, his symptoms resolved, and no cardiac events were observed at the nine-month follow‑up. The timeline of the case is shown in Figure 9.

**Figure 9:**
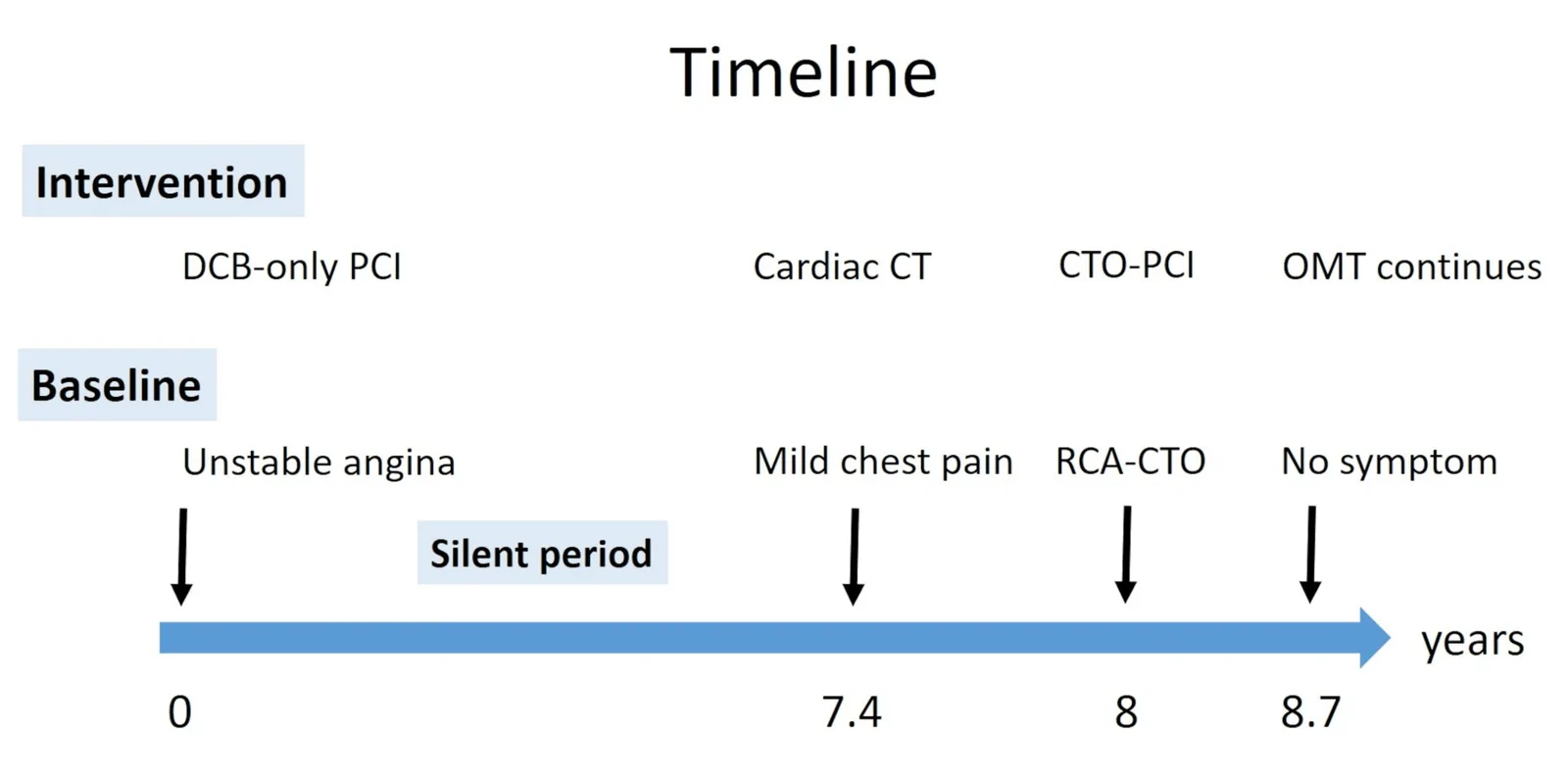
Timeline The timeline illustrates the eight-year follow-up after DCB-only PCI, showing the seven-year silent period, the development of heavy calcification on CT at 7.4 years, and the subsequent diagnosis and treatment of RCA-CTO. DCB: drug-coated balloon; PCI: percutaneous coronary intervention; CTO: chronic total occlusion; RCA: right coronary artery; OMT: optimal medical therapy; CT: computed tomography

## Discussion

This case illustrates a very late CTO that developed eight years after stentless PCI. Although mid‑term outcomes of DCB angioplasty for de novo small lesions have been reported [7], long‑term results, particularly beyond 5-8 years, and reports of very late total occlusion after DCB‑only PCI remain scarce. A stentless strategy was selected for the diffuse long mid‑RCA lesion to avoid the long‑term risks associated with permanent implants. Since its introduction, this approach has gained broader acceptance and has been applied even in selected cases of acute coronary syndrome, with favorable outcomes reported [8]. This report provides morphological characterization and mechanistic interpretation of a heavily calcified CTO at a previously treated site.

First, cardiac CT demonstrated plaque progression with severe calcification, and IVUS revealed homogeneous fibrocalcific plaque with dense calcification and high plaque burden. No thrombus‑like density was observed on CT, and IVUS did not show echolucent zones, layered morphology, pedunculated structures, or microchannels typically associated with organized thrombus [9]. These findings are more compatible with long‑term fibrocalcific progression rather than thrombotic occlusion, consistent with previously reported loose fibrous tissue‑driven CTO formation [10]. However, prior thrombus organization cannot be definitively excluded because sequential imaging was lacking. Mild progression in other coronary segments suggests contributions from both localized and generalized atherosclerotic mechanisms. Calcification progression is common in diabetes mellitus and typically requires several years to develop [10], although the exact timing of occlusion could not be determined due to lack of serial imaging.

Second, the fibrocalcific progression appears multifactorial, involving native atherosclerotic remodeling, cumulative cardiovascular risk exposure, procedural factors, and possibly drug‑related effects. Although the acute angiographic result of DCB angioplasty fulfilled conventional angiographic criteria, TIMI 3 flow without dissection or thrombus and with residual stenosis <30%, the final minimal lumen area and plaque burden were 2.67 mm² and 66.7%. IVUS demonstrated a substantial residual plaque burden. Limited acute luminal gain relative to the 2.5 mm reference vessel raised concern for subsequent lumen loss. Although DCB angioplasty is generally associated with late lumen enlargement and favorable vascular healing [3], the suboptimal acute result may have contributed to progressive fibrocalcific change in this segment.

Finally, heavy calcification during the chronic phase was consistent with the paclitaxel‑treated segment. Although paclitaxel's antiproliferative effect is considered to diminish within months [4], preclinical studies suggest that low residual tissue concentrations may persist and influence vascular remodeling, including smooth muscle cell proliferation and matrix metalloproteinase activity [11]. Local high‑density paclitaxel platforms have been associated with chronic inflammatory cell infiltration and subsequent calcification formation [12]. Calcified in-stent restenosis was often observed after DES implantation. However, device-related neoatherosclerosis is not applicable in this case because no scaffold was implanted. Residual paclitaxel may have contributed to vascular calcification; however, this possibility remains speculative in an individual case.

This case highlights that silent, heavily calcified CTO can occur even after stentless PCI, particularly in patients with diffuse coronary disease and multiple cardiovascular risk factors. Although routine cardiac CT angiography after DCB angioplasty is not recommended [13] and, in this case, we did not perform scheduled lesion follow-up using any imaging modality, very late follow‑up imaging may provide insights into long‑term vascular remodeling in selected high‑risk patients. Achieving adequate acute luminal gain during stentless PCI remains essential, as suboptimal initial results may predispose the treated segment to progressive fibrocalcific change. Aggressive secondary prevention is crucial to mitigate native atherosclerotic progression after stentless interventions.

This report has limitations. It describes a single case with long-term follow-up after DCB treatment of a de novo lesion, which inherently restricts generalizability and does not allow any inference regarding a causal relationship between DCB treatment, paclitaxel exposure, and subsequent CTO development. Gray‑scale IVUS was used, whereas optical coherence tomography would have provided higher‑resolution tissue characterization. Furthermore, histopathological assessment was not available. Finally, because the patient declined further coronary evaluation during the asymptomatic interval, morphological changes could not be assessed.

The central illustration is shown in Figure 10.

**Figure 10:**
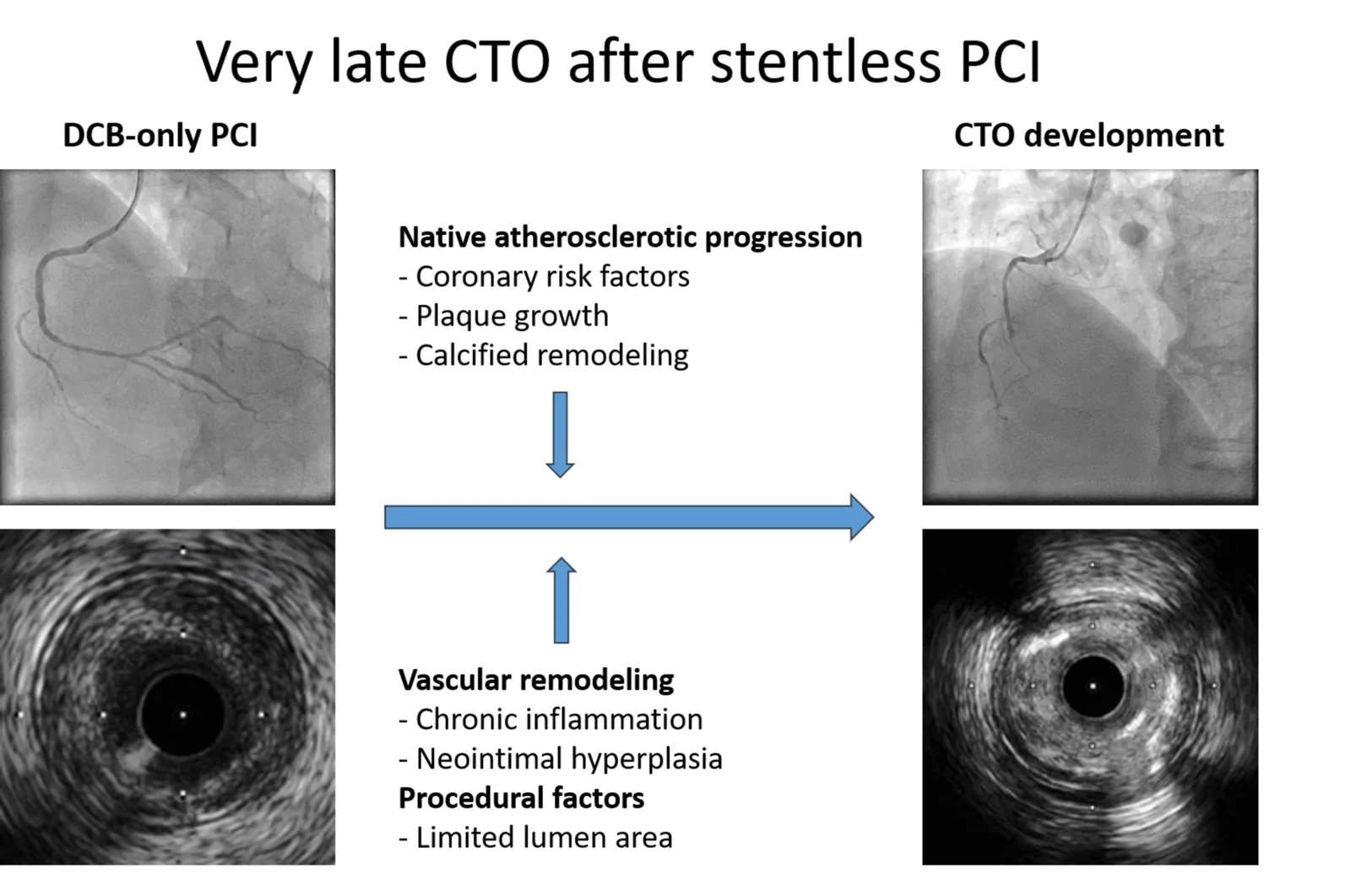
Central illustration The figure depicts the multimodal morphological features associated with a very late fibrocalcific CTO observed in a vessel previously treated with a DCB‑only strategy. CTO: chronic total occlusion; PCI: percutaneous coronary intervention; DCB: drug-coated balloon

## Conclusions

This case describes a CTO identified eight years after stentless PCI with a DCB and demonstrates how multimodality imaging, including cardiac CT and IVUS, can characterize very late lesion morphology. Cardiac CT showed severe luminal narrowing with dense calcification, while IVUS revealed homogeneous fibrocalcific plaque without thrombotic features. These complementary findings delineate a late-phase vascular morphology that may be encountered after stentless PCI in patients with diffuse disease and multiple risk factors and underscore the value of multimodality imaging when very late vascular events are clinically suspected.
